# Generating three-color pulses in high-gain harmonic-generation free-electron lasers with a tilted electron bunch

**DOI:** 10.1107/S1600577519009317

**Published:** 2019-08-12

**Authors:** Zhouyu Zhao, Heting Li, Weiwei Li, Qika Jia, Shimin Jiang, Lin Wang

**Affiliations:** a National Synchrotron Radiation Laboratory, University of Science and Technology of China, Hefei 230029, People’s Republic of China

**Keywords:** three-color, corrugated structure, tilted bunch, TGU, NTG, HGHG

## Abstract

A multi-color light source is a significant tool for nonlinear optics experiments, pump–dump/repump–probe experiments and in other fields. Here, a novel method is proposed to create three-color pulses based on a high-gain harmonic-generation (HGHG) free-electron laser with a tilted electron bunch. In this method, the initial bunch tilt is created by transverse wakefields after the bunch passes through a corrugated structure with an off-axis orbit, and is further enlarged in a following drift section. Then the tilted bunch experiences the off-axis field of a quadrupole magnet to cool down the large transverse velocity induced before. After that, it enters an HGHG configuration adopting a transverse gradient undulator (TGU) as the radiator, where only three separated fractions of the tilted bunch will resonate at three adjacent harmonics of the seed wavelength and are enabled to emit three-color pulses simultaneously. In addition, the use of the natural transverse gradient of a normal planar undulator instead of the TGU radiator to emit three-color pulses is also studied in detail. Numerical simulations including the generation of the tilted bunch and the free-electron laser radiation confirm the validity and feasibility of this scheme both for the TGU radiator and the natural gradient in the extreme-ultraviolet waveband.

## Abstract 2

A novel method is proposed to create three-color free-electron laser pulses based on high-gain harmonic generation with a tilted electron bunch which is created by a corrugated structure, a drift section and a quadrupole. Theoretical analyses and numerical simulations confirm the validity and feasibility of this scheme both for the TGU radiator and the natural gradient in the extreme-ultraviolet waveband.

## 1. Introduction   

The last decade has seen the explosive development of the free-electron laser (FEL) as one of the most promising light sources at extreme-ultraviolet and X-ray wavelengths (Ackermann *et al.*, 2007 ▸; Emma *et al.*, 2010 ▸; Ishikawa *et al.*, 2012 ▸; Allaria *et al.*, 2013*a* ▸; Kang *et al.*, 2017 ▸; Weise & Decking, 2017 ▸; Milne *et al.*, 2017 ▸), reviewed by Feng & Deng (2018 ▸). Pulses delivered by a FEL are characterized by high intensity, short wavelength and narrow bandwidth. These unique capabilities open the door to explore various scientific fields, such as molecular reaction dynamics, atomic physics, biological structures, that originate from any similar mechanisms that involve motions of electrons, molecules and other microstructure. In order to further satisfy the requirements of different experiments, FELs with various features have been widely studied and put into practice. One of the most important formats is to create FEL pulses that contain different spectral lines with time separation, namely, multi-color FELs. Benefiting from its good qualities, a multi-color FEL can be used to carry out pump–probe experiments, where the first pulse excites a sample and the second pulse is used for probing the sample after an adjustable delay time. Currently, FEL facilities with a multi-color operation mode, such as FLASH (Feldhaus, 2010 ▸; Vogt *et al.*, 2013 ▸), SACLA (Hara *et al.*, 2013 ▸), SPARC (Petrillo *et al.*, 2013 ▸; Ronsivalle *et al.*, 2014 ▸), FERMI (Allaria *et al.*, 2013*b* ▸; Ferrari *et al.*, 2016 ▸; Penco *et al.*, 2018 ▸) and LCLS (Marinelli *et al.*, 2015 ▸; Lutman *et al.*, 2016 ▸; Guetg *et al.*, 2018 ▸), are mainly focused on the generation of a two-color FEL. Except for combining FEL pulses with synchrotron radiation or other conventional light sources, these specialized three-color FEL pulses or those containing more colors are scarce. However, many FEL users require multi-color pulses, such as for pump–dump/repump–probe experiments, non­linear optics experiments and so on, and some of them have special demands: for example, multiwave mixing requires a sample to interact with multi-color ultrashort pulses of coherent radiation, *etc.* (Glover *et al.*, 2012 ▸; Bradler *et al.*, 2013 ▸; Bencivenga *et al.*, 2015 ▸; Zhang *et al.*, 2015 ▸; Mincigrucci *et al.*, 2018 ▸).

Some existing methods for generating a two-color FEL also have the potential to create pulses with more separated spectral lines. Nevertheless, generating more-color pulses may make these methods much more complicated, such as demanding a higher number of different wavelengths of the seed lasers, a higher number of different energies of the electron bunches or a higher number of different strengths of the undulator sections, which may increase the cost and technical requirements considerably. Thus a simple and economic way to achieve a multi-color FEL remains in strong demand in the short-wavelength region.

We have also proposed a new method to make two fractions of the ideally tilted bunch satisfy different resonance conditions along the whole bunch and emit two pulses at the two adjacent harmonics of the seed wavelength with a TGU radiator in the high-gain harmonic-generation (HGHG) FEL (Zhao *et al.*, 2018 ▸). In this method, each pulse can achieve its own saturation with an easily tuned energy ratio between the two pulses, and the two pulses both have ultrashort time duration of dozens of femtoseconds with a precisely controlled delay of hundreds of femtoseconds. However, to generate three-color pulses with this scheme, a much larger bunch tilt and/or a much larger field gradient of the radiator are required. This was once thought of as very challenging in practice.

In this work, we push on with this method for the generation of a three-color FEL. We focus on the generation and optimization of the tilted electron bunch, as well as the generation of the three-color pulses. The large bunch tilt is mainly created by transverse wakefields in a corrugated structure when the initial bunch passes through it in an off-axis propagation path, and is further enlarged in a following drift section. Such a tilted bunch has a large transverse velocity and its tilt amplitude is not constant when compared with that of the previous paper (Zhao *et al.*, 2018 ▸), where we utilize a linearly tilted bunch that is actually not easy to obtain in practice. Then a quadrupole magnet is used to suppress the transverse velocity induced in the corrugated structure to make the bunch travel through the HGHG configuration at a suitable transverse velocity. Subsequently, the tilted bunch is modulated by a single seed laser and dispersed by a magnetic chicane. Then, in the TGU radiator, the electrons at different transverse positions will experience different magnetic fields. If the bunch tilt and the field gradient of the radiator are large enough, there will be three fractions of the bunch resonating at three adjacent harmonics of the seed wavelength and emitting three-color ultrashort pulses.

The rest of this paper is organized as follows. We describe how to create a large tilted bunch with small transverse velocity in Section 2, and in Section 3 three-color FEL generation in a TGU radiator is presented. We also consider the possibility of fulfilling this scheme with the natural field gradient of a normal planar undulator instead of the TGU radiator in Section 4, and finally summarize in Section 5.

## 2. Generation of a large tilted bunch   

**Figure 1:**
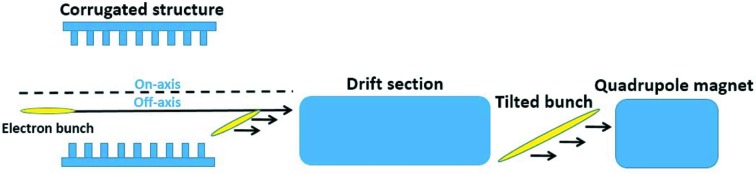
Schematic of the proposed scheme for tilted bunch generation.

A corrugated structure is widely known to cancel the bunch energy chirp introduced by a linac accelerator (Emma *et al.*, 2014 ▸). Recent studies show that it can also be used to correlate the longitudinal position with the transverse position of the electrons when the electron bunch passes off-axis through a corrugated structure, as sketched in Fig. 1 ▸. For a uniformly distributed electron bunch passing through a horizontal corrugated structure far from the axis, assuming a driving electron at (*x*_0_, *y*_0_) and a trailing electron at (*x*, *y*), with |*x* − *x*_0_| small compared with the gap size, the short-range transverse point wakes can be approximately given by (Bane *et al.*, 2016 ▸; Seok *et al.*, 2018 ▸)
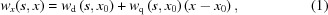


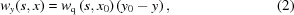
where *w*_d_(*s*, *x*_0_) and *w*_q_(*s*, *x*_0_) are the dipole and quadrupole wake functions on the offset *x*_0_, respectively.

The dipole wake offers a transverse momentum kick to create a bunch tilt with focusing in *y* and defocusing in *x* introduced by the quadrupole wake. Both wakes are proportional to the gap size, as ∝ *a* ^−4^.

Subsequently, through convolving the point wakes with the longitudinal bunch shape λ(*s*), the wakes along the whole bunch can be given as
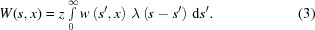
To obtain a large bunch tilt in a short corrugated structure, the gap size should be tuned to a small value. Parameters of the proposed corrugated structure are as follows: nominal half aperture *a* = 3 mm, period *p* = 0.5 mm, depth *h* = 0.6 mm, longitudinal gap *t* = 0.25 mm, plate width *w* = 20 mm, plate length *L* = 0.8 m. Due to the corrugation parameters are much smaller than the gap size (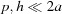), but with *h* ≥ *p* the transverse wakes at the origin in *s* (at *s* = 0^+^) start out as approximately parabolic functions of *s* from zero. The wakes at the head of the bunch are negligible but the tail experiences strong wakes. If the bunch orbit offset increases and becomes comparable with the gap size, higher-order analytical formulas or simulations also becomes necessary. In this feasibility study, we calculate the wakes including the ‘zero order’ and the ‘first order’ wakes to ensure the accuracy of the calculation, as described by Bane *et al.* (2016 ▸).

According to the wake theory above, we numerically studied the bunch evolution in the corrugated structure in three dimensions. Parameters of the electron bunch are given in Table 1 ▸. For an electron bunch with uniform distribution, the longitudinal wake strength is approximately linear. The voltage induced by a longitudinal wake at the bunch tail can be approximated as 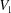 ≃ 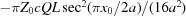, with *Z*_0_ = 377 Ω. In our case, the longitudinal voltage at the bunch tail equals −6.3 MV and then introduces an energy chirp of 1.6% without any change in the slice energy spread of the bunch. Though such an energy chirp has little influence on our three-color FEL scheme, both the longitudinal and transverse wakes are included in the simulations.

**Figure 2:**
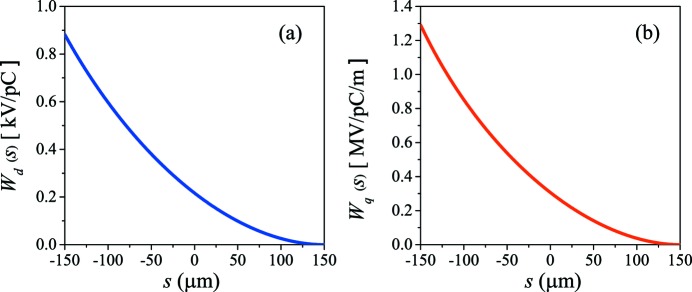
Bunch wakes of the corrugated structure for a uniform beam: (*a*) the dipole bunch wake *W*_d_(*s*) and (*b*) the quadrupole bunch wake *W*_q_(*s*), with *a* = 3 mm and *x*_0_ = 2.0 mm.

**Figure 3:**
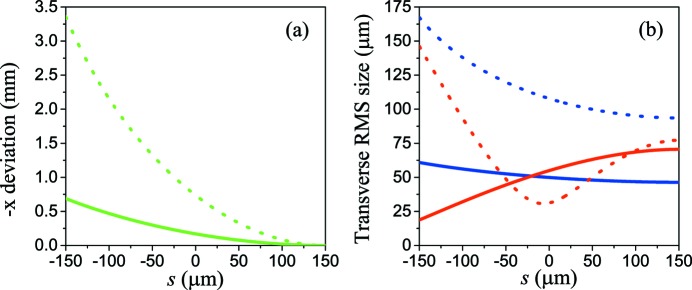
(*a*) Bunch deviation of *x* and (*b*) transverse RMS size. Solid lines: after the bunch passes through the corrugated structure. Dotted lines: after the bunch passes through the corrugated structure and the next drift section. The green line is the deviation of *x*, the blue line is σ_*x*_ and the red line is σ_*y*_.

The transverse bunch wakes are shown in Fig. 2 ▸. With an offset *x*_0_ = 2.0 mm, the tail of the bunch experiences a dipole wake of 0.9 kV pC^−1^ and a quadrupole wake of 1.3 MV pC^−1^ m^−1^. Both dipole wake and quadrupole wake contribute to the growth of the bunch tilt in the horizontal direction. As shown in Fig. 3 ▸, the wakes introduce a horizontal deviation of 0.7 mm after the bunch passes through the corrugated structure. Obviously such a tilt will increase the projected emittance of the bunch; nevertheless, the slice emittance is almost constant and thus does not influence the final FEL performance. Non-negligible issues are the introduced horizontal velocity of the bunch combined with the increase of the horizontal beam size. In the vertical direction, the bunch will suffer a transverse focusing introduced by the quadrupole wake and thus decrease the vertical beam size. In order to further increase the bunch tilt without increasing the transverse velocity of the electrons, a 1.25 m drift section is adopted after the corrugated structure. According to Fig. 3 ▸, the horizontal deviation is increased from 0.7 mm to 3.4 mm, with an inevitable increase of the beam size which may degrade the FEL performance to some extent.

The tilted bunch with a large transverse velocity will spread quickly in the transverse direction and may degrade the interaction with the seed laser in the following HGHG section. Therefore a quadrupole magnet is used to reduce the transverse velocity. When an electron with a transverse velocity *v*_0_ passes through a quadrupole magnet with an offset orbit *x*, its transverse velocity at the exit can be given as
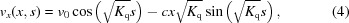
where *K*_q_ is the focusing strength.

**Figure 4:**
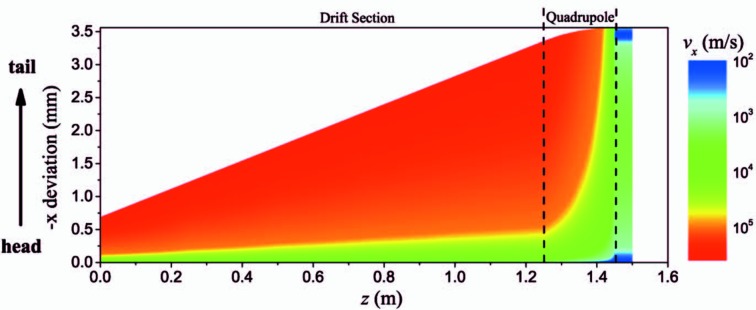
Variations of the bunch deviation at *x* and the transverse velocity along *z* when the bunch passes through the drift section and quadrupole.

For a tilted bunch generated by the corrugated structure, *v*_0_ is proportional to *x*. Thus the electron with a large transverse velocity will experience a large focusing in the quadrupole. With the careful optimization of the length and strength of the quadrupole, the transverse velocity may be greatly suppressed. As shown in Fig. 4 ▸, after passing through a quadrupole with a length of 0.2 m and an on-axis gradient of about 4 T m^−1^, the transverse velocity of the tail of the bunch is decreased from 10^5^ to 10^2^ m s^−1^. The transverse velocity of the electrons is effectively cooling down.

## 3. Generation of three-color pulses in HGHG FEL with a TGU radiator   

**Figure 5:**
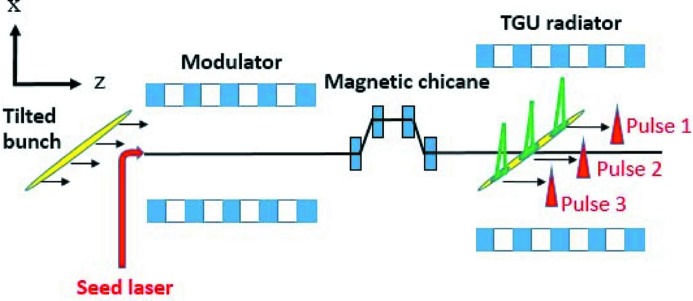
Schematic of the proposed scheme for the generation of three-color FEL pulses.

Fig. 5 ▸ shows a schematic of the proposed scheme for the generation of three-color FEL pulses. The tilted bunch is firstly modulated by a seed laser in the modulator. Due to the relative large beam size of the tilted bunch, the transverse size of the seed laser in the modulator should also be large enough to introduce an adequate energy modulation in each part of the electron bunch. Then, after the dispersive chicane, the energy modulation is converted into density modulation, and bunching at harmonics along the whole bunch is generated. The coherent synchrotron radiation (CSR) effect is not included in the simulations, but we have theoretically estimated the CSR effect in the chicane and the CSR-induced energy spread seems very small.

Then in the TGU radiator with a normalized field parameter *K*(*x*) = *K*_0_(1 + α*x*) (Huang *et al.*, 2012 ▸), where *K*_0_ is the field strength parameter on the axis of the TGU and α is the field gradient, different parts of the electron bunch will see different undulator magnetic fields. According to the FEL resonance condition, the wavelength of the radiation emitted from each part of the bunch is
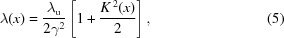
where γ is the Lorentz factor of the electrons and λ_u_ is the period of the radiator. However, only the radiation at the harmonics of the seed laser will be coherently amplified because the electron beam is bunched at these discrete wavelengths.

Assuming the generation of three-color pulses at three adjacent harmonics, the resonant undulator parameters *K*_1_, *K*_2_, *K*_3_ for these three harmonics can be calculated from the above equation. The time separations among the three FEL pulses are determined by the longitudinal positions of the three bunch fractions that emit the coherent radiation. According to Fig. 3(*a*) ▸, the bunch tilt created in Section 2 can be approximately regarded as a cubic dependence on *s* and can be fitted as η(*s*) = *x*[mm]/*s*[mm] = −9.8 + 80*s* − 180*s* ^2^. Thus the time separation Δ*t* = Δ*s*/*c* can be further calculated as
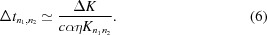
Here Δ*K* is the difference between the two resonant harmonics and 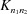 is the average *K* value of the *n*_1_th and *n*_2_th harmonics.

We have carried out the three-dimensional time-dependent numerical simulations to verify the effectiveness of the proposed method. The main parameters refer to the Dalian Coherent Light Source (DCLS), which is an extreme-ultraviolet FEL user facility (Zhang *et al.*, 2013 ▸). Energy modulation in the modulator was performed using *Genesis* 1.3 code (Reiche, 1999 ▸) and phase space evolution in the magnetic chicane was tracked using *Elegant* code (Borland, 2000 ▸).

The tilted bunch used in the simulations is described in the above section. As shown in Fig. 5 ▸, the tilted bunch travels parallel along the normal modulator axis in which a seed laser of 240 nm is used to modulate the bunch. The modulator consists of five periods with period length of 50 mm. To ensure the adequate energy modulation in each part of the large tilted bunch, a seed laser with a beam waist of 2.3 mm is adopted. Next, a dispersion section converts the energy modulation into a density modulation to form the strong bunching at harmonics.

According to equation (5), at higher harmonics, Δ*K* between these harmonics is smaller in the radiator. Thus it would be helpful to carry out this multi-color FEL scheme at high harmonics since the requirement on the product αη can be reduced. However, in an HGHG FEL, the harmonic order cannot be too large. Therefore, the harmonic orders should be selected in a moderate range to achieve multi-color pulses with considerable FEL power. In our case, we consider generating three-color pulses at the fourth, fifth and sixth harmonics of the seed laser. The period length of the TGU radiator is 30 mm, thus Δ*K* equals 0.58 for the fourth and sixth harmonics. The undulator strength parameter resonant at these three harmonics are *K*_1_ = 1.7, *K*_2_ = 1.39 and *K*_3_ = 1.13, respectively. The undulator strength parameter on the axis should be chosen around the center of *K*_1_ and *K*_3_ to cover the total target harmonics. Here, we set *K*_0_ = 1.49. The transverse field gradient of the TGU radiator α = −150 m^−1^ makes *K*(*x*) vary from 1.88 to 1.01 while the *x* deviation of the proposed tilted bunch varies from −1.75 to 1.75 mm. Therefore the tilted bunch can cover the three resonant conditions at harmonics and emit three-color FEL pulses. Each pulse is independent and can reach saturation in a single radiator. The other non-resonant electrons will experience self-amplified spontaneous emission and contribute little to the total output power. Because of the high quality of the electron bunch and low harmonic orders converted from the seed laser, a short radiator is adopted which is composed of 50 periods. Parameters of the modulator and the radiator are listed in Table 2 ▸.

**Figure 6:**
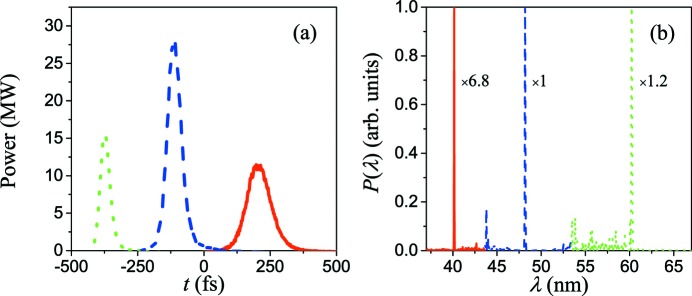
Time structure (*a*) and spectrum (*b*) of three-color FEL pulses at the exit of the TGU radiator, in which the dotted green line represents 60 nm, the dashed blue line represents 48 nm and the solid red line represents 40 nm.

The simulation results of three-color FEL pulses at the exit of the TGU radiator are given in Fig. 6 ▸. Due to the non­uniform energy modulation of the bunch, the bunching factor at the middle of the bunch is greater than that at the two sides, thus the peak power of the fifth harmonic is greater than the other two pulses. For the 60 nm pulse (fourth harmonic), 48 nm pulse (fifth harmonic) and 40 nm pulse (sixth harmonic), the peak powers are 16 MW, 28 MW and 12 MW, respectively. As given in Fig. 3 ▸, as the tilt amplitude along the bunch increases rapidly from head to tail, it is easy to figure out that the fractions that satisfy resonance at different harmonics will become shorter gradually, thus leading to the shortening of the time duration of three pulses from head to tail. The corresponding FWHM time duration of the sixth, fifth and fourth harmonics are 105 fs, 61 fs and 45 fs, respectively. The time separation between the adjacent pulses is about 306 fs and 262 fs, while those calculated by equation (6) are about 330 fs and 280 fs, respectively. These three-color pulses with such time structure have great potential to carry out relevant experiments directly.

In the spectral field, these three pulses are all close to the Fourier transform limit; the FWHM bandwidth of each pulse is about 0.2%. However, the fast-growing beam size and the tilt amplitude at the tail of the bunch will cause some deviation of the resonant position and thus result in the generation of a small burr in the spectrum. Nevertheless, such a burr has little influence on the total performance of the three-color FEL pulses.

**Figure 7:**
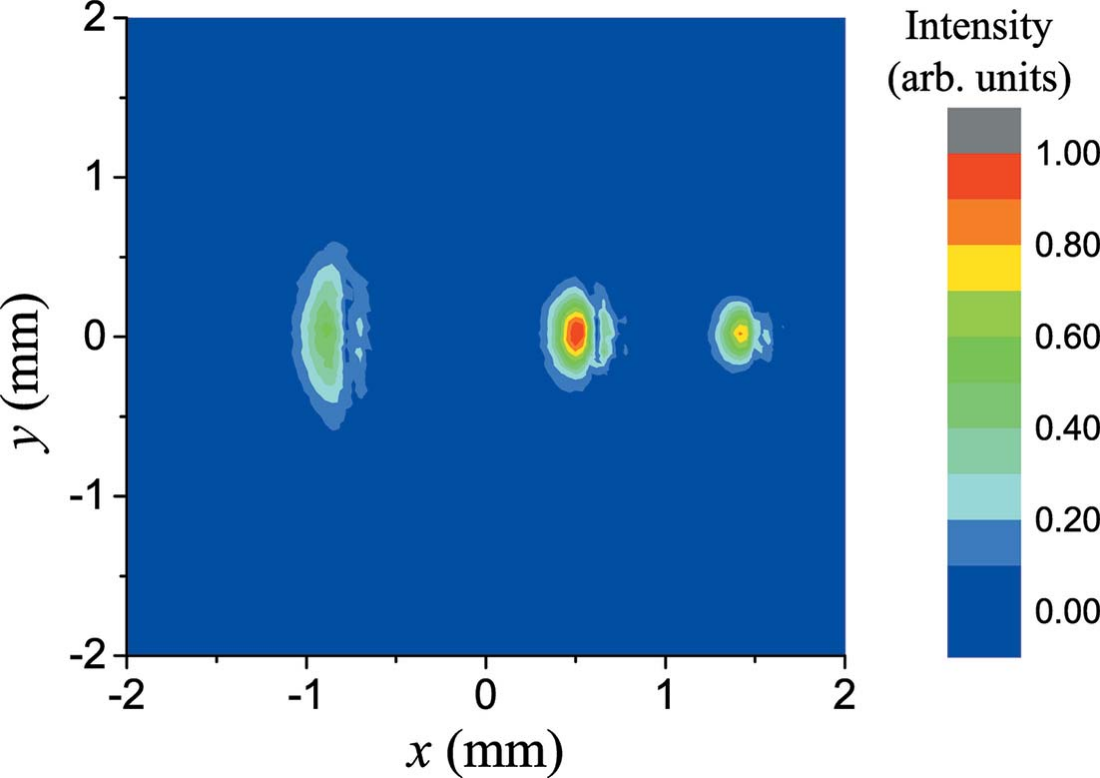
Transverse distribution of three-color FEL pulses, in which the spot on the left-hand side represents 60 nm, that in the middle represents 48 nm and that on the right-hand side represents 40 nm.

Fig. 7 ▸ shows the transverse distribution of three-color FEL pulses, in which three separated spots can be seen on the screen. The pulses on the two sides are close to being symmetric about the middle pulse with transverse separations of about 1.4 mm and 0.8 mm. Generally, these three-color pulses should be focused on the same position of the sample in most relevant experiments. To achieve such a spatial overlap of these pulses, a carefully designed reflecting mirror with toroidal surface is the potential solution to compensate for the spatial separation by introducing an optical path difference.

It is worth pointing out that, in the above simulations, the generation of the three harmonic pulses is difficult to do in a single run because of the limited covering range of the wavelength in *Genesis*. Since the three harmonics are generated from three separated fractions of the electron bunch, these three radiation processes have little impact on each other. Thus three-color FEL simulations in the radiator were performed in three separate runs and each run focused on one harmonic of the seed laser.

## 4. Possibility of fulfilling this scheme with the natural field gradient of a normal undulator   

In the above section, generation of three-color FEL pulses with a TGU radiator is studied in detail. However, to produce such three-color pulses requires a specially designed TGU with a large transverse gradient, which is not easy to realize and tune (Bernhard *et al.*, 2018 ▸). Thus we propose to use the natural transverse gradient (NTG) of a normal planar undulator instead of the TGU radiator.

**Figure 8:**
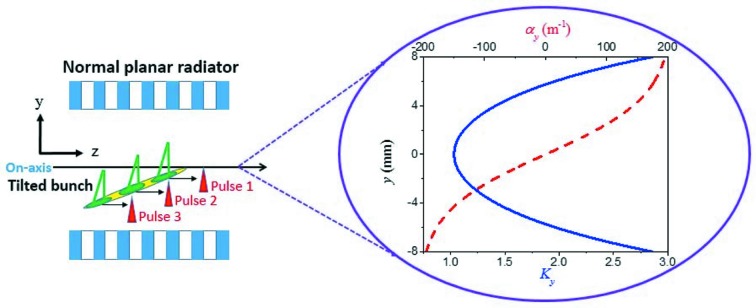
Left: schematic of the proposed scheme for three-color FEL generation in a normal planar radiator. Right: variation of the undulator field parameter (solid blue line) and the natural transverse gradient (dashed red line) in the vertical direction.

In a normal planar undulator placed as shown in Fig. 8 ▸, the undulator field parameter *K* and the natural vertical gradient α_*y*_ along the *y* direction can be described as (Jia & Li, 2017 ▸; Zhao *et al.*, 2017 ▸)
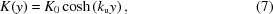


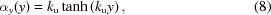
where *k*_u_ is the wavenumber of the undulator field. From these equations we can see that, for a normal planar undulator with fixed period length, *K* and α_*y*_ depend on the off-axis position of the bunch.

Taking the above parameters as an example, the undulator field is shown in the right-hand figure of Fig. 8 ▸. Compared with the usual TGU used in Section 3, the natural gradient is not constant but grows rapidly as the vertical offset increases. Considering injecting the tilted bunch into the normal undulator with the head on the axis, the head of the bunch will experiences a small gradient. Under this condition, the tilted bunch should be long enough in the vertical direction to cover these three resonant conditions to realize this scheme. The initial bunch tilt in the vertical direction is created by a vertical corrugated structure similar to the horizontal one. With a longer drift section of 3 m and the same parameters of the corrugated structure described in Section 2, we obtain a tilted bunch with a vertical position from head to tail of Δ*y* = 7 mm but with the same longitudinal bunch length. Actually this bunch tilt can also be achieved by adjusting the bunch offset in the corrugated structure and keeping the length of the drift section unchanged.

Following the modulator and dispersive section with the same parameters, the bunching at harmonics is generated. However, compared with the *x*-tilted bunch scheme, the magnetic field of the modulator in this scheme should be changed to the horizontal direction to avoid the large variation of the undulator parameter *K* along the bunch. This will guarantee that all electrons from head to tail are resonant at the seed frequency and avoid the unwanted natural focusing which may degrade the FEL performance in the following radiator. The polarization of the seed laser also needs to be changed to the vertical direction. In the normal radiator, the period number and the period length are also the same as with the TGU radiator. For the NTG scheme, the choice on the harmonic orders is similar to that of the TGU scheme. The on-axis undulator parameter is set to be *K*_0_ = 1.03, which is smaller than that in the TGU scheme. Obviously, the range of *K*_*y*_ can satisfy the conditions for generating three-color FEL pulses for the above tilted bunch. If the bunch is placed away from the axis, *K*_0_ can be reduced. Thus the NTG scheme has the potential of working at an open gap of the radiator.

**Figure 9:**
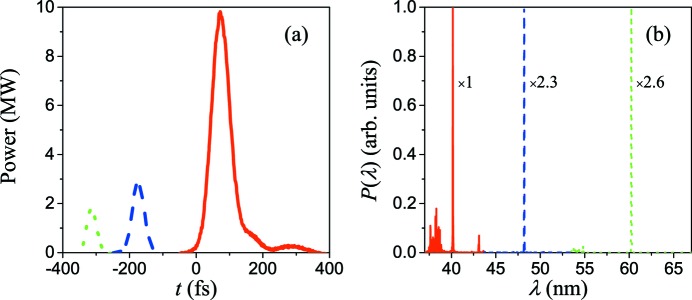
Time structure (*a*) and spectrum (*b*) of three-color FEL pulses at the exit of the normal planar radiator, in which the dotted green lines represent 60 nm, the dashed blue lines represent 48 nm and the solid red lines represent 40 nm.

Fig. 9 ▸ shows the time structure and spectrum of the generated three-color pulses. These pulses have separated spectral lines and a peak power of several megawatts. The power reduction of each pulse compared with that generated in the TGU radiator is mainly because here the tilted bunch has a larger global transverse size so that the beam current in each transverse slice becomes about half as low. If the users want to keep the FEL power at the same level, the total charge needs to be increased. The FWHM pulse width of the sixth, fifth and fourth harmonics are about 60 fs, 45 fs and 30 fs, respectively, and the time separation between the adjacent pulses is about 260 fs and 140 fs.

Note that the bunch passing through the undulator in an off-axis orbit will suffer a weak betatron oscillation. However, in our scheme, the betatron wavelength λ_β*y*_ = 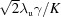 ≃ 15 m is far longer than the undulator length of 1.5 m, thus the natural focusing effect can be neglected.

**Figure 10:**
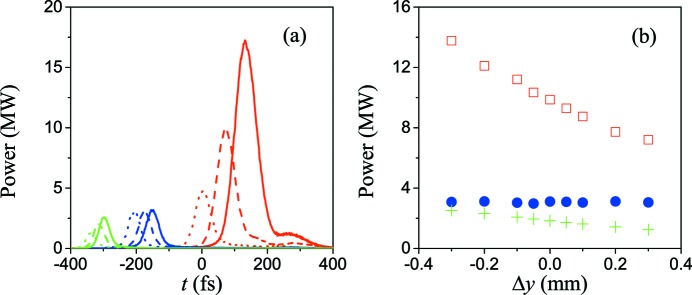
(*a*) Variation of the profile of three-color FEL pulses with the undulator field parameter on the axis in the normal planar radiator, in which solid lines represent *K*_0_ = 1.06, dashed lines represent *K*_0_ = 1.03 and dotted lines represent *K*_0_ = 0.99. (*b*) Variation of the three-color FEL power with the offset of the vertical bunch orbit in a normal planar radiator, in which Δ*y* is the deviation from the orbit of *y* = 0 mm at the head of the bunch. Green crosses represent 60 nm, blue circles represent 48 nm and red squares represent 40 nm.

The main difference in using the natural gradient of a normal undulator from a TGU is that the gradient varies with the coordinate of the bunch-tilted direction. As shown in Fig. 10(*a*) ▸, with increasing *K*_0_ the power of the fourth and fifth harmonic changes very slightly, but on the other hand the power of the sixth harmonic changes a lot. Such a phenomenon is the result of α_*y*_ increasing slowly around the head but rapidly at the tail of the bunch, so that the variation of the position of the bunch fraction resonant at higher harmonic is larger than that at lower harmonics as well as the variation of the bunching factors. In addition, with decreasing *K*_0_, the resonant fractions move away from the axis and will see a large gradient, leading to the shortening of the time duration of the three pulses. The dependence of the power on the bunch orbit offset from the head to the axis is exhibited in Fig. 10(*b*) ▸. One can find that the power of these three pulses is almost not changed when varying the bunch head around the axis. If the variation of the power remains at less than 5%, the shake and the accuracy of the orbit control should be better than 0.1 mm, which can be easily satisfied by the current technology of beam measurement and control.

In general, the generation of a three-color FEL based on the natural gradient has a good tolerance not only on the undulator field parameter but also on the bunch orbit offset. Since the gradient can be easily tuned by varying the orbit offset of the bunch, it also has the feature of a good adjustability. This may be very helpful for carrying on the relevant demonstration experiment for the generation of a three-color FEL.

## 5. Summary   

We have presented a new method of generating three-color FEL pulses based on the HGHG configuration driven by a tilted bunch in the extreme-ultraviolet waveband. The creation of a large bunch tilt with an acceptable beam quality relies upon the combination of a corrugated structure, a drift section and a quadrupole magnet. Detailed analysis and three-dimensional simulations validated the feasibility of generating the required tilted bunch. Combined with a TGU radiator in the HGHG configuration, such a tilted bunch is able to generate three-color pulses at three adjacent harmonics of the seed laser. Further study demonstrated that three-color pulses can also be achieved with the natural gradient of a normal undulator with this scheme, and the radiation power of the three pulses has good tolerances on the undulator field parameter and the bunch orbit offset.

With this method, only one electron bunch, one seed laser and one radiator section are required. Based on the large bunch tilt and the field gradient of the radiator, three-color ultrashort pulses with large frequency separations and time delay of a few hundred femtoseconds can be obtained without splitting the radiator into several subsections. This method is worthwhile in that it can provide an extension of the FEL properties for existing seeded FEL facilities to offer three-color pulses for some special users. However, the ratio of the three frequencies is almost fixed. The three frequencies and their gaps can be tuned by changing the seed frequency and the setting of the harmonic conversion numbers, but only in a range that is not very large. The time delay of the three pulses can be tuned on the level from several tens of femtoseconds to about a few hundred femtoseconds for practical electron beams; however, the ratio of the delay between the three pulses can only be changed in a very limited range.

## Figures and Tables

**Table 1:** Parameters of the electron bunch

Parameter	Specification	Units
Energy, *E*	400	MeV
Energy spread, σ_γ_	200	keV
Normalized emittance, ∊_n_	1	mm mrad
Bunch transverse size, σ_*x*_/σ_*y*_	30/70	µm
Full bunch length, *l*_b_	1	ps
Bunch current, *I*	800	A

**Table 2:** Parameters of undulators

Parameter	Specification	Units
Modulator period length	50	mm
Modulator period number	5	–
Modulator parameter, *K*_m_	3.28	–
Radiator period length	30	mm
Radiator period number	50	–
Radiator parameter on the axis, *K*_0_	1.49	–
Radiator strength gradient, α	−150	m^−1^

## References

[bb1] Ackermann, W., Asova, G., Ayvazyan, V., Azima, A., Baboi, N., Bähr, J., Balandin, V., Beutner, B., Brandt, A., Bolzmann, A., Brinkmann, R., Brovko, O. I., Castellano, M., Castro, P., Catani, L., Chiadroni, E., Choroba, S., Cianchi, A., Costello, J. T., Cubaynes, D., Dardis, J., Decking, W., Delsim-Hashemi, H., Delserieys, A., Di Pirro, G., Dohlus, M., Düsterer, S., Eckhardt, A., Edwards, H. T., Faatz, B., Feldhaus, J., Flöttmann, K., Frisch, J., Fröhlich, L., Garvey, T., Gensch, U., Gerth, Ch., Görler, M., Golubeva, N., Grabosch, H.-J., Grecki, M., Grimm, O., Hacker, K., Hahn, U., Han, J. H., Honkavaara, K., Hott, T., Hüning, M., Ivanisenko, Y., Jaeschke, E., Jalmuzna, W., Jezynski, T., Kammering, R., Katalev, V., Kavanagh, K., Kennedy, E. T., Khodyachykh, S., Klose, K., Kocharyan, V., Körfer, M., Kollewe, M., Koprek, W., Korepanov, S., Kostin, D., Krassilnikov, M., Kube, G., Kuhlmann, M., Lewis, C. L. S., Lilje, L., Limberg, T., Lipka, D., Löhl, F., Luna, H., Luong, M., Martins, M., Meyer, M., Michelato, P., Miltchev, V., Möller, W. D., Monaco, L., Müller, W. F. O., Napieralski, O., Napoly, O., Nicolosi, P., Nölle, D., Nuñez, T., Oppelt, A., Pagani, C., Paparella, R., Pchalek, N., Pedregosa-Gutierrez, J., Petersen, B., Petrosyan, B., Petrosyan, G., Petrosyan, L., Pflüger, J., Plönjes, E., Poletto, L., Pozniak, K., Prat, E., Proch, D., Pucyk, P., Radcliffe, P., Redlin, H., Rehlich, K., Richter, M., Roehrs, M., Roensch, J., Romaniuk, R., Ross, M., Rossbach, J., Rybnikov, V., Sachwitz, M., Saldin, E. L., Sandner, W., Schlarb, H., Schmidt, B., Schmitz, M., Schmüser, P., Schneider, J. R., Schneidmiller, E. A., Schnepp, S., Schreiber, S., Seidel, M., Sertore, D., Shabunov, A. V., Simon, C., Simrock, S., Sombrowski, E., Sorokin, A. A., Spanknebel, P., Spesyvtsev, R., Staykov, L., Steffen, B., Stephan, F., Stulle, F., Thom, H., Tiedtke, K., Tischer, M., Toleikis, S., Treusch, R., Trines, D., Tsakov, I., Vogel, E., Weiland, T., Weise, H., Wellhöfer, M., Wendt, M., Will, I., Winter, A., Wittenburg, K., Wurth, W., Yeates, P., Yurkov, M. V., Zagorodnov, I. & Zapfe, K. (2007). *Nat. Photon.* **1**, 336–342.

[bb14] Allaria, E., Bencivenga, F., Borghes, R., Capotondi, F., Castronovo, D., Charalambous, P., Cinquegrana, P., Danailov, M. B., De Ninno, G., Demidovich, A., Di Mitri, S., Diviacco, B., Fausti, D., Fawley, W. M., Ferrari, E., Froehlich, L., Gauthier, D., Gessini, A., Giannessi, L., Ivanov, R., Kiskinova, M., Kurdi, G., Mahieu, B., Mahne, N., Nikolov, I., Masciovecchio, C., Pedersoli, E., Penco, G., Raimondi, L., Serpico, C., Sigalotti, P., Spampinati, S., Spezzani, C., Svetina, C., Trovò, M. & Zangrando, M. (2013*b*). *Nat. Commun.* **4**, 2476. 10.1038/ncomms3476 PMC3791458 24048228

[bb4] Allaria, E., Castronovo, D., Cinquegrana, P., Craievich, P., Dal Forno, M., Danailov, M. B., D’Auria, G., Demidovich, A., De Ninno, G., Di Mitri, S., Diviacco, B., Fawley, W. M., Ferianis, M., Ferrari, E., Froehlich, L., Gaio, G., Gauthier, D., Giannessi, L., Ivanov, R., Mahieu, B., Mahne, N., Nikolov, I., Parmigiani, F., Penco, G., Raimondi, L., Scafuri, C., Serpico, C., Sigalotti, P., Spampinati, S., Spezzani, C., Svandrlik, M., Svetina, C., Trovo, M., Veronese, M., Zangrando, D. & Zangrando, M. (2013*a*). *Nat. Photon.* **7**, 913–918.

[bb27] Bane, K., Stupakov, G. & Zagorodnov, I. (2016). *Phys. Rev. Accel. Beams*, **19**, 084401.

[bb22] Bencivenga, F., Cucini, R., Capotondi, F., Battistoni, A., Mincigrucci, R., Giangrisostomi, E., Gessini, A., Manfredda, M., Nikolov, I. P., Pedersoli, E., Principi, E., Svetina, C., Parisse, P., Casolari, F., Danailov, M. B., Kiskinova, M. & Masciovecchio, C. (2015). *Nature*, **520**, 205–208. 10.1038/nature14341 PMC4413025 25855456

[bb33] Bernhard, A., Afonso Rodríguez, V., Kuschel, S., Leier, M., Peiffer, P., Sävert, A., Schwab, M., Werner, W., Widmann, C., Will, A., Müller, A. & Kaluza, M. (2018). *Nucl. Instrum. Methods Phys. Res. A*, **909**, 391–397.

[bb32] Borland, M. (2000). ANL Technical Report LS-287. Argonne National Laboratory, Argonne, Illinois, USA.

[bb21] Bradler, M., Werhahn, J., Hutzler, D., Fuhrmann, S., Heider, R., Riedle, E., Iglev, H. & Kienberger, R. (2013). *Opt. Express*, **21**, 20145–20158. 10.1364/OE.21.020145 24105560

[bb2] Emma, P., Akre, R., Arthur, J., Bionta, R., Bostedt, C., Bozek, J., Brachmann, A., Bucksbaum, P., Coffee, R., Decker, F., Ding, Y., Dowell, D., Edstrom, S., Fisher, A., Frisch, J., Gilevich, S., Hastings, J., Hays, G., Hering, P., Huang, Z., Iverson, R., Loos, H., Messerschmidt, M., Miahnahri, A., Moeller, S., Nuhn, H., Pile, G., Ratner, D., Rzepiela, J., Schultz, D., Smith, T., Stefan, P., Tompkins, H., Turner, J., Welch, J., White, W., Wu, J., Yocky, G. & Galayda, J. (2010). *Nat. Photon.* **4**, 641–647.

[bb26] Emma, P., Venturini, M., Bane, K., Stupakov, G., Kang, H. S., Chae, M. S., Hong, J., Min, C. K., Yang, H., Ha, T., Lee, W. W., Park, C. D., Park, S. J. & Ko, I. S. (2014). *Phys. Rev. Lett.* **112**, 034801. 10.1103/PhysRevLett.112.034801 24484143

[bb9] Feldhaus, J. (2010). *J. Phys. B At. Mol. Opt. Phys.* **43**, 194002.

[bb8] Feng, C. & Deng, H. (2018). *Nucl. Sci. Tech.* **29**, 160.

[bb15] Ferrari, E., Spezzani, C., Fortuna, F., Delaunay, R., Vidal, F., Nikolov, I., Cinquegrana, P., Diviacco, B., Gauthier, D., Penco, G., Ribič, P. R., Roussel, E., Trovò, M., Moussy, J. B., Pincelli, T., Lounis, L., Manfredda, M., Pedersoli, E., Capotondi, F., Svetina, C., Mahne, N., Zangrando, M., Raimondi, L., Demidovich, A., Giannessi, L., De Ninno, G., Danailov, M. B., Allaria, E. & Sacchi, M. (2016). *Nat. Commun.* **7**, 10343. 10.1038/ncomms10343 PMC4735510 26757813

[bb20] Glover, T., Fritz, D., Cammarata, M., Allison, T. K., Coh, S., Feldkamp, J. M., Lemke, H., Zhu, D., Feng, Y., Coffee, R. N., Fuchs, M., Ghimire, S., Chen, J., Shwartz, S., Reis, D. A., Harris, S. E. & Hastings, J. B. (2012). *Nature*, **488**, 603–608. 10.1038/nature11340 22932384

[bb19] Guetg, M. W., Lutman, A., Ding, Y., Maxwell, T. J. & Huang, Z. (2018). *Phys. Rev. Lett.* **120**, 264802. 10.1103/PhysRevLett.120.264802 30004747

[bb11] Hara, T., Inubushi, Y., Katayama, T., Sato, T., Tanaka, H., Tanaka, T., Togashi, T., Togawa, K., Tono, K., Yabashi, M. & Ishikawa, T. (2013). *Nat. Commun.* **4**, 2919. 10.1038/ncomms3919 24301682

[bb29] Huang, Z., Ding, Y. & Schroeder, C. (2012). *Phys. Rev. Lett.* **109**, 204801. 10.1103/PhysRevLett.109.204801 23215493

[bb3] Ishikawa, T., Aoyagi, H., Asaka, T., Asano, Y., Azumi, N., Bizen, T., Ego, H., Fukami, K., Fukui, T., Furukawa, Y., Goto, S., Hanaki, H., Hara, T., Hasegawa, T., Hatsui, T., Higashiya, A., Hirono, T., Hosoda, N., Ishii, M., Inagaki, T., Inubushi, Y., Itoga, T., Joti, Y., Kago, M., Kameshima, T., Kimura, H., Kirihara, Y., Kiyomichi, A., Kobayashi, T., Kondo, C., Kudo, T., Maesaka, H., Maréchal, X. M., Masuda, T., Matsubara, S., Matsumoto, T., Matsushita, T., Matsui, S., Nagasono, M., Nariyama, N., Ohashi, H., Ohata, T., Ohshima, T., Ono, S., Otake, Y., Saji, C., Sakurai, T., Sato, T., Sawada, K., Seike, T., Shirasawa, K., Sugimoto, T., Suzuki, S., Takahashi, S., Takebe, H., Takeshita, K., Tamasaku, K., Tanaka, H., Tanaka, R., Tanaka, T., Togashi, T., Togawa, K., Tokuhisa, A., Tomizawa, H., Tono, K., Wu, S., Yabashi, M., Yamaga, M., Yamashita, A., Yanagida, K., Zhang, C., Shintake, T., Kitamura, H. & Kumagai, N. (2012). *Nat. Photon.* **6**, 540–554.

[bb34] Jia, Q. & Li, H. (2017). *Phys. Rev. Accel. Beams*, **20**, 020707.

[bb5] Kang, H. S., Min, C. K., Heo, H., Kim, C., Yang, H., Kim, G., Nam, I., Baek, S. Y., Choi, H. J., Mun, G., Park, B. R., Suh, Y. J., Shin, D. C., Hu, J., Hong, J., Jung, S., Kim, S. H., Kim, K., Na, D., Park, S. S., Park, Y. J., Han, J. H., Jung, Y. G., Jeong, S. H., Lee, H. G., Lee, S., Lee, S., Lee, W. W., Oh, B., Suh, H. S., Parc, Y. W., Park, S. J., Kim, M. H., Jung, N. S., Kim, Y. C., Lee, M. S., Lee, B. H., Sung, C. W., Mok, I. S., Yang, J. M., Lee, C. S., Shin, H., Kim, J. H., Kim, Y., Lee, J. H., Park, S. Y., Kim, J., Park, J., Eom, I., Rah, S., Kim, S., Nam, K. H., Park, J., Park, J., Kim, S., Kwon, S., Park, S. H., Kim, K. S., Hyun, H., Kim, S. N., Kim, S., Hwang, S. M., Kim, M. J., Lim, C. Y., Yu, C. J., Kim, B. S., Kang, T. H., Kim, K. W., Kim, S. H., Lee, H. S., Lee, H. S., Park, K. H., Koo, T. Y., Kim, D. E. & Ko, I. S. (2017). *Nat. Photon.* **11**, 708–713.

[bb18] Lutman, A. A., Maxwell, T. J., MacArthur, J. P., Guetg, M. W., Berrah, N., Coffee, R. N., Ding, Y., Huang, Z., Marinelli, A., Moeller, S. & Zemella, J. C. U. (2016). *Nat. Photon.* **10**, 745–750.

[bb17] Marinelli, A., Ratner, D., Lutman, A., Turner, J., Welch, J., Decker, F. J., Loos, H., Behrens, C., Gilevich, S., Miahnahri, A. A., Vetter, S., Maxwell, T. J., Ding, Y., Coffee, R., Wakatsuki, S. & Huang, Z. (2015). *Nat. Commun.* **6**, 6369. 10.1038/ncomms7369 PMC4366525 25744344

[bb7] Milne, C., Schietinger, T., Aiba, M., Alarcon, A., Alex, J., Anghel, A., Arsov, V., Beard, C., Beaud, P., Bettoni, S., Bopp, M., Brands, H., Brönnimann, M., Brunnenkant, I., Calvi, M., Citterio, A., Craievich, P., Csatari Divall, M., Dällenbach, M., D’Amico, M., Dax, A., Deng, Y., Dietrich, A., Dinapoli, R., Divall, E., Dordevic, S., Ebner, S., Erny, C., Fitze, H., Flechsig, U., Follath, R., Frei, F., Gärtner, F., Ganter, R., Garvey, T., Geng, Z., Gorgisyan, I., Gough, C., Hauff, A., Hauri, C., Hiller, N., Humar, T., Hunziker, S., Ingold, G., Ischebeck, R., Janousch, M., Juranić, P., Jurcevic, M., Kaiser, M., Kalantari, B., Kalt, R., Keil, B., Kittel, C., Knopp, G., Koprek, W., Lemke, H., Lippuner, T., Llorente Sancho, D., Löhl, F., Lopez-Cuenca, C., Märki, F., Marcellini, F., Marinkovic, G., Martiel, I., Menzel, R., Mozzanica, A., Nass, K., Orlandi, G., Ozkan Loch, C., Panepucci, E., Paraliev, M., Patterson, B., Pedrini, B., Pedrozzi, M., Pollet, P., Pradervand, C., Prat, E., Radi, P., Raguin, J., Redford, S., Rehanek, J., Réhault, J., Reiche, S., Ringele, M., Rittmann, J., Rivkin, L., Romann, A., Ruat, M., Ruder, C., Sala, L., Schebacher, L., Schilcher, T., Schlott, V., Schmidt, T., Schmitt, B., Shi, X., Stadler, M., Stingelin, L., Sturzenegger, W., Szlachetko, J., Thattil, D., Treyer, D., Trisorio, A., Tron, W., Vetter, S., Vicario, C., Voulot, D., Wang, M., Zamofing, T., Zellweger, C., Zennaro, R., Zimoch, E., Abela, R., Patthey, L. & Braun, H. (2017). *Appl. Sci.* **7**, 720.

[bb24] Mincigrucci, R., Foglia, L., Naumenko, D., Pedersoli, E., Simoncig, A., Cucini, R., Gessini, A., Kiskinova, M., Kurdi, G., Mahne, N., Manfredda, M., Nikolov, I. P., Principi, E., Raimondi, L., Zangrando, M., Masciovecchio, C., Capotondi, F. & Bencivenga, F. (2018). *Nucl. Instrum. Methods Phys. Res. A*, **907**, 132–148.

[bb16] Penco, G., Allaria, E., Bassanese, S., Cinquegrana, P., Cleva, S., Danailov, M. B., Demidovich, A., Ferianis, M., Gaio, G., Giannessi, L., Masciovecchio, C., Predonzani, M., Rossi, F., Roussel, E., Spampinati, S. & Trovò, M. (2018). *New J. Phys.* **20**, 053047.

[bb12] Petrillo, V., Anania, M., Artioli, M., Bacci, A., Bellaveglia, M., Chiadroni, E., Cianchi, A., Ciocci, F., Dattoli, G., Di Giovenale, D., Di Pirro, G., Ferrario, M., Gatti, G., Giannessi, L., Mostacci, A., Musumeci, P., Petralia, A., Pompili, R., Quattromini, M., Rau, J. V., Ronsivalle, C., Rossi, A. R., Sabia, E., Vaccarezza, C. & Villa, F. (2013). *Phys. Rev. Lett.* **111**, 114802. 10.1103/PhysRevLett.111.114802 24074094

[bb31] Reiche, S. (1999). *Nucl. Instrum. Methods Phys. Res. A*, **429**, 243–248.

[bb13] Ronsivalle, C., Anania, M., Bacci, A., Bellaveglia, M., Chiadroni, E., Cianchi, A., Ciocci, F., Dattoli, G., Di Giovenale, D., Di Pirro, G., Ferrario, M., Gatti, G., Giannessi, L., Mostacci, A., Musumeci, P., Palumbo, L., Petralia, A., Petrillo, V., Pompili, R., Rau, J. V., Rossi, A. R., Vaccarezza, C. & Villa, F. (2014). *New J. Phys.* **16**, 033018.

[bb28] Seok, J., Chung, M., Kang, H., Min, C. & Na, D. (2018). *Phys. Rev. Accel. Beams*, **21**, 022801.

[bb10] Vogt, M., Faatz, B., Feldhaus, J., Honkavaara, K., Schreiber, S. & Treusch, R. (2013). *Proceedings of the Fourth International Particle Accelerator Conference (IPAC2013)*, 12–17 May 2013, Shanghai, China, pp. 1167–1169. TUPEA004.

[bb6] Weise, H. & Decking, W. (2017). *Proceedings of the 38th International Free Electron Laser Conference (FEL2017)*, 20–25 August 2017, Santa Fe, NM, USA, pp. 9–13. MOC03.

[bb30] Zhang, T., Deng, H., Zhang, W., Wu, G., Dai, D., Wang, D., Yang, X. & Zhao, Z. (2013). *Chin. Phys. C.* **37**, 118101.

[bb23] Zhang, Z., Lambrev, P., Wells, K., Garab, G. & Tan, H. S. (2015). *Nat. Commun.* **6**, 7914. 10.1038/ncomms8914 PMC4532882 26228055

[bb35] Zhao, Z., Li, H. & Jia, Q. (2017). *J. Synchrotron Rad.* **24**, 906–911. 10.1107/S1600577517008402 28862611

[bb25] Zhao, Z., Li, H. & Jia, Q. (2018). *Phys. Rev. Accel. Beams*, **21**, 020701.

